# The UK Pregnancies Better Eating and Activity Trial (UPBEAT); Pregnancy Outcomes and Health Behaviours by Obesity Class

**DOI:** 10.3390/ijerph17134712

**Published:** 2020-06-30

**Authors:** Lucy Peacock, Paul T. Seed, Kathryn V. Dalrymple, Sara L. White, Lucilla Poston, Angela C. Flynn

**Affiliations:** Department of Women and Children’s Health, School of Life Course Sciences, King’s College London, 10th Floor North Wing, St Thomas’ Hospital, Westminster Bridge Road, London SE1 7EH, UK; lucy.peacock@kcl.ac.uk (L.P.); paul.seed@kcl.ac.uk (P.T.S.); kathryn.dalrymple@kcl.ac.uk (K.V.D.); sara.white@kcl.ac.uk (S.L.W.); lucilla.poston@kcl.ac.uk (L.P.)

**Keywords:** pregnant women with obesity, obesity class, antenatal lifestyle intervention, gestational weight gain, dietary intake

## Abstract

The effectiveness of antenatal intervention in women with increasing obesity is unknown. This study investigated whether there was a differential effect of antenatal intervention on diet, physical activity and pregnancy outcomes in women stratified by obesity class using data from the UK Pregnancies Better Eating and Activity Trial (UPBEAT) (*n* = 1555). The stratification was by World Health Organization classifications: Class I, II and III (30–34.9 kg/m^2^, 35–39.9 kg/m^2^ and ≥40 kg/m^2^). Using linear and logistic regression, adjusted for confounders, outcomes were assessed post-intervention (27^+0^–28^+6^ weeks’ gestation) and in late pregnancy (34^+0^–36^+0^ weeks’ gestation). Interactions between obesity class and the intervention were explored. Compared to the standard care arm, class III intervention women had lower gestational weight gain (GWG) (−1.87 kg; 95% CI −3.29 to −0.47, *p* = 0.009), and the effect of the intervention was greater in class III compared to class I, by −2.01 kg (95% CI −3.45 to −0.57, *p* = 0.006). Class I and II intervention women reported significantly lower dietary glycaemic load and saturated fat intake across their pregnancy. This differential effect of the intervention suggests antenatal interventions for women with obesity should stratify outcomes by obesity severity. This would inform evidence-based antenatal strategies for high-risk groups, including women with a BMI ≥ 40 kg/m^2^.

## 1. Introduction

Obesity in pregnancy is associated with adverse maternal and fetal outcomes [1]. An ‘umbrella’ description of all women with a BMI ≥ 30 kg/m^2^ as ‘obese’ may neglect the effects of increasing BMI above this threshold in relation to pregnancy risk, in addition to differences in the propensity to benefit from antenatal interventions. The National Maternity and Perinatal Audit, which utilises the WHO BMI classes [2], reports that in England, 13% of women at the time of booking their antenatal appointment fall within Class I (BMI of 30–34.9 kg/m^2^), 5% within Class II (35–39.9 kg/m^2^) and 2% within Class III (≥40 kg/m^2^) obesity [3].

A gradient of risk exists for the incidence of gestational diabetes (GDM) [4], pre-eclampsia [5] and large for gestational age (LGA) infants [6], as obesity severity increases in pregnancy. Furthermore, gestational weight gain (GWG) is an additional risk factor for pregnancy complications, demonstrating strong associations with LGA and macrosomic infants [7]. The National Academy of Medicine (NAM) (previously the Institute of Medicine) recommends a lower GWG range for all women with obesity of 5–9 kg [8]. These guidelines do not differentiate by obesity severity, attributed to a lack of evidence regarding maternal and newborn outcomes. There is some evidence to suggest that weight gain compatible with improved pregnancy outcomes is lower with increasing obesity class [9]; however, observational studies are inconsistent regarding an optimal GWG range for obese women. Weight gain below the NAM recommendations has been shown to be associated with an increased incidence of small for gestational age (SGA) infants, and although this risk is smaller in those with a BMI ≥ 40 kg/m^2^ [10], large-scale studies suggest the association remains for obesity classes I, II and III [11]. There is a need for robust evidence that quantifies GWG ranges for women with increasing severity of obesity.

The maternal diet is often targeted in interventions to reduce GWG, and has implications for pregnancy outcomes, alongside future maternal and offspring health [12]. Despite its potential as a modifiable risk factor, there is a paucity of research on the diet of pregnant women with obesity, although there are reports of suboptimal quality [13], with inadequate carbohydrate and excessive saturated fat intake [14]. Additionally, pregnant women in obesity Class III have been shown to consume an energy rich diet, deficient in key micronutrients, compared to women with a BMI < 25 kg/m^2^ [15]. This is of concern as weight gain in pregnancy compatible with the NAM guidelines has been observed in women with obesity in whom energy intake (EI) is less than energy expenditure [16]. There is a critical need for more information on the dietary intake of pregnant women with increasing obesity severity, in order to create informed nutritional guidelines for this high-risk group.

Antenatal lifestyle interventions have demonstrated modest reductions in GWG and improved dietary intake in women with a BMI ≥ 25 kg/m^2^ [17]. However, the reporting of pregnancy outcomes and health behaviours via obesity severity has seldom been attempted, and the validity of results when applied to women of different classes of BMI remains unquantified. This study aimed to determine the effectiveness of an antenatal intervention into the health behaviours and pregnancy outcomes in women with increasing obesity severity, using data from a large randomised controlled trial, the UK Pregnancies Better Eating and Activity trial (UPBEAT). Measures of both nutritional intake and physical activity were evaluated by obesity class in each arm of the trial, in addition to pregnancy outcomes. We have previously reported that the primary outcomes of reductions of GDM and LGA infants were not achieved, although the intervention group as a whole showed improved dietary intake, increased physical activity and reduced GWG, as well as maternal adiposity [18].

## 2. Materials and Methods 

### 2.1. Study Design

UPBEAT was a multicentre, randomised controlled trial across 8 UK sites (London (three centres), Bradford, Glasgow, Manchester, Newcastle and Sunderland), and was approved by the NHS research ethics committee (UK integrated research application system, reference 09/H0802/5). Women over 16 years with a BMI ≥ 30 kg/m^2^, a singleton pregnancy and gestational age between 15^+0^ and 18^+6^ weeks’ gestation, in the absence of any underlying disease, were eligible for inclusion. Following written informed consent, allocation to the control or intervention arm was undertaken by computer generated randomisation, and minimised by ethnicity (Black, White, Asian, other), parity (primiparous, multiparous), age (≤24, 25–29, 30–34, ≥35 years), BMI (30.0–34.9, 35.0–39.9, ≥40 kg/m^2^) and centre [19].

### 2.2. UPBEAT Intervention

The UPBEAT intervention consisted of an initial interview with a health-trainer and a further eight weekly individual or group-based sessions of 1 to 1.5 h. The sessions addressed approaches to achieving goals related to the study aims. The dietary component of the UPBEAT intervention encouraged a healthier eating pattern without energy restriction. Dietary advice aimed to reduce glycaemic load (GL) and saturated fat intake. To reduce GL, participants were encouraged to exchange carbohydrate rich foods, such as bread, rice and potatoes, with a high glycaemic index (GI), for a low-GI version, and reduce the consumption of sugar sweetened beverages including fruit juice. To reduce saturated fat intake, the selection of dairy products and snacks with a lower saturated fat content was encouraged and a reduction in the intake of fatty meats and meat products was recommended [19]. To increase physical activity, advice focused on increasing daily step counts and being more active in daily life. This was individually tailored depending on goals set by intervention participants, however the objective was to encourage an incremental increase in walking from study entry, with focus on walking at a moderate intensity. Materials provided to intervention participants included a DVD of an exercise regimen appropriate for pregnancy, a pedometer and a log book for recording weekly goals. Pedometers were used for motivational and monitoring purposes only. The standard care arm of the trial entailed attending routine appointments at the trial centre, as per local practice.

### 2.3. Data Collection

Data were collected at three visits: study entry (15^+0^–18^+6^ weeks’ gestation), post-intervention (27^+0^–28^+6^ weeks’ gestation) and late gestation (34^+0^–36^+0^ weeks’ gestation). Study entry demographic data included age (years), BMI (kg/m^2^), ethnicity (Black, White, Asian, other), parity (nulliparous, multiparous), smoking status (smoker, ex-smoker, non-smoker), level of relative deprivation (Index of Multiple Deprivation quintiles [IMD]; scores were calculated for the region of residence), and highest educational attainment. Diet was assessed in all participants using a food frequency questionnaire (FFQ) adapted from the UK arm of the European Prospective Investigation into Cancer Study [20]. The list of food items was accompanied by a multiple response grid, and frequency of food consumed was estimated over the preceding month. An automated program transformed FFQ data into nutrient intakes. Participants estimated as under-reporting (≤4·5 MJ/day) and over-reporting (≥20.0 MJ/day) energy intake were excluded [20,21]. Physical activity was assessed using the International Physical Activity Questionnaire (IPAQ) [22]. In all participants, a 75 g oral glucose tolerance test (OGTT) was undertaken at 27^+0^ to 28^+6^ weeks gestation, and diagnosis of GDM was made in accordance with International Association of Diabetes and Pregnancy Study Groups (IADPSG) criteria [23]. If GDM was diagnosed, women were referred for routine management in their locality.

### 2.4. Outcomes

This analysis utilises primary and secondary outcomes identical to those selected in the original UPBEAT study [18]. As such, the primary maternal outcome was a reduction in GDM and for the infant a reduction in the incidence of LGA (≥90th customised birthweight centile). Additional maternal outcomes included fasting plasma blood glucose, 1 h and 2 h venous blood glucose, pre-eclampsia and caesarean section. Anthropometric outcomes included gestational weight (kg) gained from the women’s weight at study entry −1.25 kg, to 27^+0^ to 28^+6^ weeks’ gestation, and to 34^+0^–36^+0^ weeks’ gestation (taken to be total weight gained), as well as sum of skinfold thicknesses (calculated by the addition of biceps, triceps, suprailiac and subscapular skinfold thicknesses (mm)).

Dietary outcomes were total EI (kcal/day), GI, GL, carbohydrate (%E), protein (%E), total fat (%E), fibre intake (g/day) and saturated fat (%E) intake. Physical activity outcomes were time spent walking (minutes/week) and metabolic equivalents (METs), demonstrating the ratio of energy expenditure of activity to energy expenditure at rest. For the infant, additional outcomes included macrosomia (birthweight 4 kg or more), and the incidence of SGA at ≤10th customised birthweight centile.

### 2.5. Statistical Analysis

All data from women randomised to the control and intervention group were stratified by WHO obesity classes (30.0–34.9, 35.0–39.9 and ≥40.0 kg/m^2^), respectively. An analysis of variance (ANOVA) and a chi-square test, for continuous and categorical variables, respectively, were performed on the sociodemographic data collected from the whole group at study entry. To assess the effect of the UPBEAT intervention on dietary intake, an analysis of co-variance (ANCOVA) was used to compare the respective diets of obesity classes randomised to control and intervention, adjusting for study entry values. Adjusted linear regression was used to assess the effect of the UPBEAT intervention on the continuous pregnancy outcomes, with results expressed as mean differences and 95% confidence intervals (CI), and adjusted logistic regression was applied to binary outcomes, expressed as odds ratio (OR) with 95% CIs. The models were adjusted for confounders including index of multiple deprivation, parity, age, ethnicity and years in full-time education. To determine if the impact of intervention on dietary intake and pregnancy outcomes differed by obesity class, a series of models with interaction terms was fitted, and effect size compared with likelihood ratio tests. All analysis was conducted with Stata version 13 (StataCorp, College Station, Texas, USA), and *p* < 0.05 was taken as the level of significance.

## 3. Results

### 3.1. Maternal Characteristics 

The characteristics of the UPBEAT participants by obesity class at study entry are shown in Table 1. Approximately half of the participants were in obesity Class I (*n* = 765, 49%), a third in Class II (*n* = 508, 33%) and 18% (*n* = 281) in Class III. Class III women were more likely to reside in an area of high deprivation (51.6%), compared to those of Class I (40.4%) and Class II (43.0%). Educational attainment varied by obesity class, with more participants in Class III having education extending only to, or not meeting, GCSE level. There were no differences in age, ethnicity, parity, history of GDM or living area by obesity class.

### 3.2. Dietary and Physical Activity Outcomes 

At study entry, there were no significant differences in nutrient intakes or physical activity levels by obesity class (Supplementary Table S1).

Following delivery of the UPBEAT intervention, compared to the equivalent obesity class receiving standard care, Class I and Class II participants in the intervention group achieved the dietary objectives (Table 2). Specifically, intervention participants in both classes decreased dietary GL and reduced saturated fat intake post-intervention (*p* < 0.05), and maintained this change in late pregnancy (*p* < 0.05). In addition, EI and GI were reduced, and protein intake was increased at both assessment time-points (*p* < 0.05). For intervention participants in obesity Class III, neither GL nor saturated fat intake were reduced post-intervention; however, saturated fat intake was reduced in late pregnancy (*p* < 0.05). GI was reduced and protein intake increased post-intervention and in late pregnancy (*p* < 0.05). Carbohydrate intake was reduced post-intervention (*p* < 0.05). There was a significant interaction between intervention group and obesity class for total EI assessed post-intervention (*p* = 0.02). The effect of the intervention on EI in obesity Class III was increased compared to Class I by 222 Kcal/day (95% CI 46.66 to 397.46, *p* = 0.013). 

As a measure of physical activity, time walking was increased in the obesity Class I intervention group post-intervention (0.32; 95% CI 0.14 to 0.51, *p* = 0.001), and this was maintained at 34^+0^–36^+0^ weeks’ gestation. Class I participants demonstrated significantly increased METs post-intervention (0.27; 95% CI 0.08 to 0.46, *p* = 0.006), however not in late pregnancy. Class II and III did not significantly change their levels of physical activity if randomised to the intervention group (Table 2).

### 3.3. Maternal and Neonatal Outcomes

**Figure 1:**
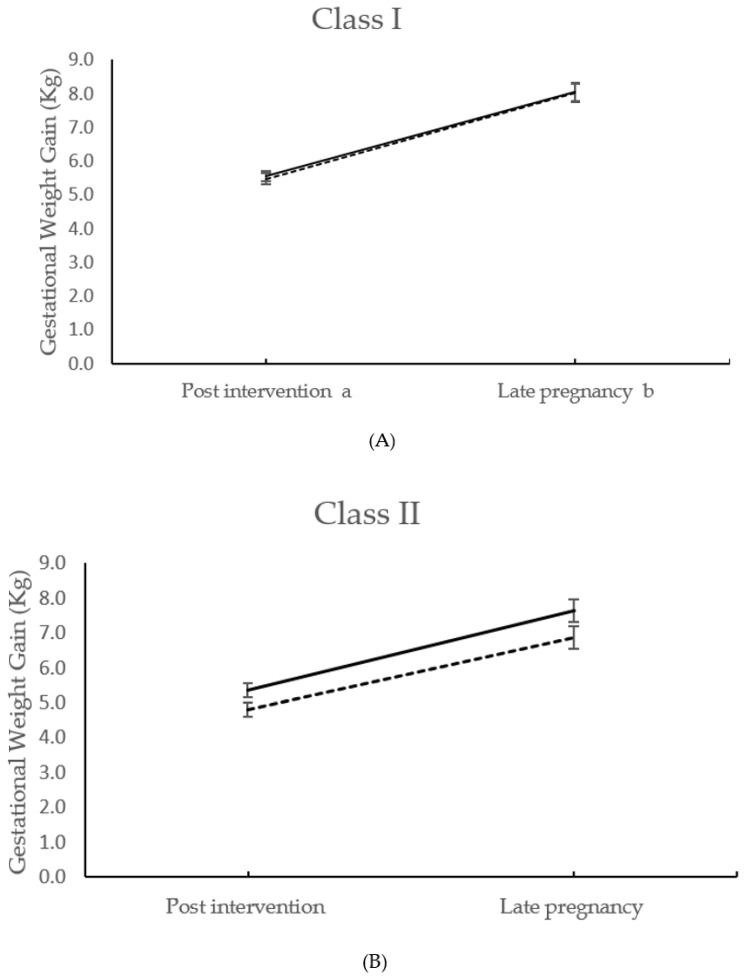

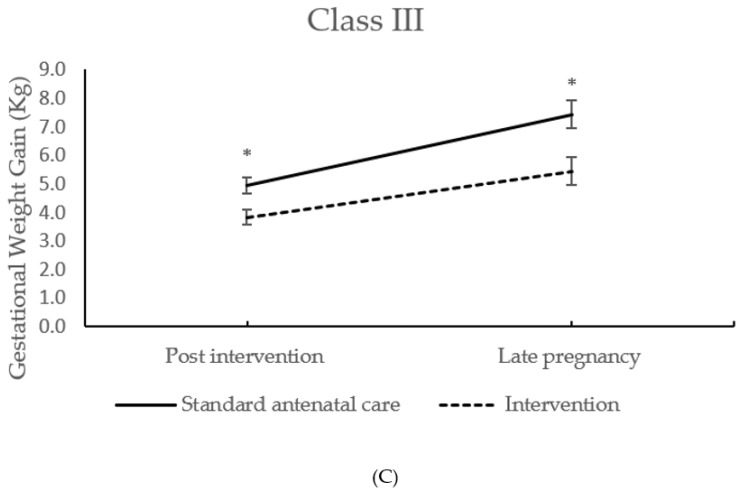
GWG at 27^+0^–28^+6^ weeks’ gestation and at 34^+0^–36^+0^ weeks’ gestation, for participants randomised to standard antenatal care and intervention groups in (**A**) obesity Class I, (**B**) obesity Class II**,** (**C**) obesity Class III. Data points are means with standard error bars. * p < 0.05 to be taken as significant.

Compared to the control arm, GWG was lower in Class III intervention participants at 28 weeks’ gestation (−1.12 kg; 95% CI −1.88 to −0.37, *p* = 0.004), which was significant following adjustment for confounders (Figure 1). This significant reduction was maintained in late pregnancy, (−1.87 kg; 95% CI −3.29 to −0.47, *p* = 0.009). There was a significant interaction between intervention group and obesity class with regards to GWG post-intervention (*p* = 0.033) and in late pregnancy (*p* = 0.023). The effect of the intervention on total GWG was increased in Class III, compared to Class I, by −2.01 kg (95% CI −3.45 to −0.57, *p* = 0.006). Class I intervention women had a significantly lower sum of skinfold thicknesses post-intervention, compared to those receiving standard care (−3.90 mm; −7.19 to −0.60, *p* = 0.021) (Table 3). For infant outcomes, there was an increased incidence of SGA infants in the Class I intervention group by population birthweight centiles (OR: 1.70; 1.06 to 2.71). All other pregnancy and neonatal outcomes were not different between treatment arms by obesity class.

## 4. Discussion

In a large group of ethnically diverse pregnant women with obesity, in which there was a high level of socio-economic deprivation, this study demonstrated that participants with a BMI ≥ 40 kg/m^2^ demonstrated a significant decrease in GWG in response to the antenatal intervention, when compared to women receiving standard antenatal care. The UPBEAT study previously reported that the intervention was associated with a reduction in GWG (−0.55 kg; 95% CI −1.08 to −0.02, *p* = 0.041) in the obese BMI class heterogenous intervention group [18], compared to the standard care arm. These data provide additional insight into and details regarding the interaction between GWG and obesity class.

As a known modifiable risk factor for adverse pregnancy outcomes [7], GWG is often targeted by antenatal intervention. The lower GWG in Class III does not appear to have impacted on the main outcomes of the trial, as stratification revealed no change in the incidence of GDM or LGA infants in all classes, including in women with a BMI ≥ 40 kg/m^2^. This is consistent with the results of a systematic review on the effects of antenatal dietary and physical activity interventions, which showed a reduction in GWG, but no significant effect of a reduction in maternal and offspring composite outcomes [24] across all BMI subgroups, including women with obesity. The evidence is conflicting concerning the appropriate amount of weight gain by obesity class, and to date, a lack of consensus exists. The combined lowest risks for SGA, LGA and caesarean section have been reported in a systematic review in women with Class III obesity who gained no weight overall during pregnancy [9]. Weight maintenance in obese pregnant women is likely to be achieved by an increase in fat mobilisation, as a study of body composition revealed that women with Class III obesity lose fat, in comparison to Class I and II women who gain fat, in the second trimester [25]. However, weight gain of <5 kg in pregnancy by women with Class III obesity has been shown to significantly increase the risk of low birth weight infants and neonatal mortality, relative to those gaining weight within the NAM limits [11]. As such, more evidence is needed from randomised controlled trials, stratifying by obesity class, to define a weight gain compatible with optimal outcomes for women with increasing obesity.

Excessive GWG consistently predicts postpartum weight retention (PPWR) [7]. A positive implication of the reduction in GWG for Class III women may therefore be a reduction in postnatal weight retention. PPWR is a risk factor for future obesity [26], and the antenatal period could represent a window where intervention can interrupt the cycle of accumulating and retaining weight for these high-risk women. Since an increase of ≥ 3 BMI units between pregnancies increases the risk of GDM, hypertensive disorders and caesarean section in the next pregnancy [27], limiting PPWR may improve maternal and neonatal outcomes in the future. Documenting longer term outcomes of antenatal intervention by obesity class is key to establishing strategies to tackle future pregnancy risk and promote maternal health.

In this study, dietary changes also differed by obesity class, with Class I and II intervention participants significantly reducing GL and saturated fat intake, and maintaining these changes into late pregnancy. The nutritional improvements reported by Class III participants randomised to the intervention may have contributed to their lower weight gain. From the results of antenatal interventions, it is difficult to determine which dietary methodologies are effective in preventing excessive GWG [17], due to their heterogeneity, e.g., low GI, low fat, low calorie intake. The macronutrient content of the diet, including fat, protein and carbohydrate intake, was not found to consistently associate with GWG in a systematic review of 46 observational studies and 10 trials, which stated it was unclear whether different macronutrients could affect weight gain independently of their energy content. Higher EI during pregnancy was however associated with GWG [28].

In obese pregnancy, the relationship between EI and GWG has recently been afforded greater clarity by a study which revealed that women with obesity, who gained the recommended weight of 5–9 kg, maintained a negative energy balance in pregnancy, as opposed to those who exceeded weight gain recommendations, in whom EI exceeded expenditure [16]. When the weight gain of those meeting recommendations was further characterised, an accumulation of fat free mass was compensated for by a loss of fat mass, leading the authors to conclude that fat mobilisation in obese women removes the need to increase caloric intake to meet the requirements of pregnancy.

Although our results show that women of Class III obesity did not decrease their EI, or increase levels of physical activity with randomisation to the intervention, it cannot be ruled out that energy balance contributed to the greater efficacy of the intervention in restricting GWG in this group. Mis-reporters of EI are more likely to be obese than plausible supporters [29], which could have led to inaccuracies in the dietary reporting from the FFQs. Furthermore, our study entry demographic data detailed that women of Class III obesity were more likely to have fewer years of education and reside in areas of higher deprivation, both of which have been found to be predictive of energy mis-reporting [29,30]. The indeterminate accuracy of reported EI in those with severe obesity limits any conclusive associations that may have otherwise been drawn between EI and GWG. Thus, as a priority, a validation of the methods of reporting EI by obesity class in pregnant women is urgently required.

The national guidance on weight management in pregnancy recommends that obese women do not diet while they are pregnant, and instead offers generic healthy eating advice [31]. This reflects that dietary requirements remain unquantified for obese pregnancy. Reporting on nutritional outcomes of antenatal intervention by obesity class is needed to understand the relationship between dietary guidance and clinical outcomes for pregnant women with obesity of increasing severity. This is particularly the case for those of Class III obesity, who have been found to exceed the NAM guidelines for weight gain in 40% of cases [32], with adjusted analysis revealing this to significantly increase the risk of severely adverse combined maternal and perinatal outcomes [33].

The study strengths include the UPBEAT study being a large, multicentre, randomised controlled trial, which included women across all obese BMI classes. Nutritional outcomes were reported, contributing to the limited evidence base describing dietary intake in obese pregnant women, and allowing further understanding of their receptivity to dietary changes in pregnancy. Women of low socio-economic status were of high prevalence, which increases the likelihood of results being valid across the general population, and therefore represents a valuable information source for public health policy making. Limitations include the use of self-reported dietary and physical activity data. In addition, it may be that there was inadequate statistical power to determine health behaviour changes and pregnancy outcomes by increasing degree of obesity severity.

## 5. Conclusions

This study highlights that the findings of antenatal interventions cannot be generalised for all pregnant women with obesity, as the pregnancy outcomes and health behaviours differed by obesity class. The greater efficacy of the UPBEAT intervention in lowering GWG in women with Class III obesity supports antenatal weight management strategies for women with severe obesity. More research, stratifying the effect of antenatal intervention on pregnancy outcomes and health behaviours by obesity class, is urgently required, to inform future strategies for improving the health of pregnant women with obesity.

## Figures and Tables

**Table 1:** Study entry characteristics of women by obesity class.

	Obesity Class			
Maternal Characteristics at Study Entry	Class I	Class II	Class III	*p* ^c^
	*n* = 765 (49.2%)	*n* = 508 (32.6%)	*n* = 281 (18.1%)	
Age	30.5 ± 5.5	30.6 ± 5.5	30.3 ± 5.4	0.700
Ethnic origin				
White	463 (60.5%)	332 (65.4%)	178 (63.4%)	0.165
Black	200 (26.1%)	121 (23.8%)	80 (28.5%)	
Asian	53 (6.9%)	27 (5.3%)	15 (5.3%)	
Other	49 (6.4%)	28 (5.5%)	8 (2.9%)	
Parity				
Nulliparous	343 (44.8%)	219 (43.1%)	112 (39.9%)	0.351
Multiparous	422 (55.2%)	289 (56.9%)	169 (60.1%)	
Previous history of				
gestational diabetes	14 (3.3%)	10 (3.5%)	8 (4.7%)	0.582
(multiparous only)				
Index of multiple				
Deprivation ^a^				
1 (least deprived)	39 (5.1%)	22 (4.3%)	4 (1.4%)	0.026
5 (most deprived)	308 (40.4%)	218 (43.0%)	144 (51.6%)	
Living area				
Inner city	470 (61.4%)	315 (62.0%)	162 (57.7%)	0.273
Suburban	271 (35.4%)	167 (32.9%)	107 (38.1%)	
Rural	24 (3.1%)	26 (5.1%)	12 (4.3%)	
Educational attainment ^b^				
None/GCSE	151(19.7%)	93 (18.3%)	73 (26.0%)	0.006
A level	115 (15.0%)	84 (16.5%)	48 (17.1%)	
Degree	333 (43.5%)	195 (38.4%)	88 (31.3%)	
Vocational qualification	166 (21.7%)	136 (26.8%)	72 (25.6%)	

Data are mean ± SD or number of participants/total (%). ^a^ Scores were calculated by region of residence. UK scores were developed with consideration of employment and income domains. Presented are the first and last quintile. ^b^ GCSE—General Certificate of Secondary Education; A-level—General Certificate of Education Advanced Level. ^c^ *p* value for differences between study entry characteristics of the obesity classes, assessed using ANOVA for continuous variables, and chi squared analysis for categorical variables. *p* < 0.05 taken as significant.

**Table 2:** Dietary Responses and Physical Activity at study entry, post-intervention and in late gestation, by intervention group and obesity class.

Nutrition		Time Points ^a^	Standard Care	Intervention	Mean Difference (95% CI)	*p* ^b^	*p* ^c^
Total energy	Class I	Study entry	1909.59 ± 614.87	1800.08 ± 588.79			
(Kcal/day)		Post-intervention	1826.53 ± 544.88	1609.32 ± 424.68	−195.97 (−275.88 to −116.07)	<0.001	
		Late pregnancy	1822.18 ± 567.35	1625.15 ± 525.40	−151.96 (−257.86 to −46.07)	0.005	
	Class II	Study entry	1857.01 ± 588.92	1788.87 ± 595.84			
		Post-intervention	1767.12 ± 559.40	1592.28 ± 435.05	−221.87 (−334.92 to −108.81)	<0.001	
		Late pregnancy	1694.79 ± 483.48	1533.63 ± 408.87	−150.41 (−250.38 to −50.44)	0.003	
	Class III	Study entry	1741.14 ± 631.59	1923.02 ± 622.16			
		Post-intervention	1749.47 ± 537.85	1801.79 ± 549.32	36.24 (−138.29 to 210.77)	0.682	
		Late pregnancy	1687.30 ± 511.29	1699.41 ± 572.24	−25.77 (−222.68 to 171.13)	0.796	
Interaction (post-intervention)						0.024	
Glycaemic index	Class I	Study entry	57.08 ± 4.40	56.61 ± 3.90			
(0–100)		Post-intervention	57.04 ± 4.11	54.07 ± 3.90	−2.78 (−3.42 to −2.14)	<0.001	
		Late pregnancy	56.81 ± 4.18	54.73 ± 4.02	−2.12 (−2.83 to −1.41)	<0.001	
	Class II	Study entry	56.58 ± 4.07	56.84 ± 4.08			
		Post-intervention	56.91 ± 3.86	54.41 ± 3.98	−2.43 (−3.21 to −1.66)	<0.001	
		Late pregnancy	56.53 ± 4.02	54.12 ± 4.02	−2.46 (−3.41 to −1.50)	<0.001	
	Class III	Study entry	56.93 ± 3.35	57.13 ± 3.62			
		Post-intervention	57.21 ± 3.50	54.73 ± 3.97	−2.29 (−3.36 to −1.21)	<0.001	
		Late pregnancy	57.32 ± 3.92	54.89 ± 4.51	−2.32 (−3.64 to −1.00)	0.001	
Glycaemic load	Class I	Study entry	146.75 ± 59.03	134.43 ± 51.17			
(per day)		Post-intervention	135.44 ± 46.19	110.49 ± 36.87	−22.58 (−29.60 to −15.57)	<0.001	
		Late pregnancy	133.86 ± 50.52	133.11 ± 41.92	−17.06 (−25.96 to −8.16)	<0.001	
	Class II	Study entry	137.18 ± 50.96	133.36 ± 50.97			
		Post-intervention	131.07 ± 49.17	109.66 ± 38.13	−24.74 (−34.54 to −14.95)	<0.001	
		Late pregnancy	122.44 ± 41.64	103.38 ± 35.11	−17.84 (−26.95 to −8.72)	<0.001	
	Class III	Study entry	130.84 ± 57.76	139.16 ± 48.19			
		Post-intervention	128.99 ± 46.39	121.10 ± 42.80	−8.96 (−22.96 to 5.05)	0.208	
		Late pregnancy	121.16 ± 37.04	114.88 ± 46.73	−9.45 (−24.26 to 5.36)	0.209	
Carbohydrate	Class I	Study entry	50.09 ± 7.61	49.26 ± 7.45			
(% energy)		Post-intervention	48.63 ± 6.69	47.57 ± 7.17	−0.91 (−2.08 to 0.26)	0.129	
		Late pregnancy	48.08 ± 7.07	47.67 ± 7.92	−0.61 (−2.08 to 0.85)	0.412	
	Class II	Study entry	48.85 ± 7.55	49.17 ± 7.00			
		Post-intervention	48.68 ± 6.60	47.21 ± 6.94	−1.66 (−3.06 to −0.25)	0.021	
		Late pregnancy	47.80 ± 6.75	46.52 ± 7.14	−0.98 (−2.59 to 0. 62)	0.230	
	Class III	Study entry	49.01 ± 6.34	47.93 ± 8.01			
		Post-intervention	48.30 ± 6.60	46.02 ± 7.74	−2.37 (−4.42 to −0.32)	0.024	
		Late pregnancy	47.45 ± 6.66	46.00 ± 7.69	−1.36 (−3.87 to 1.14)	0.282	
Protein	Class I	Study entry	19.36 ± 4.26	19.86 ± 4.43			
(% energy)		Post-intervention	19.98 ± 4.16	21.97 ± 4.45	1.78 (1.08 to 2.47)	<0.001	
		Late pregnancy	20.12 ± 4.53	21.57 ± 4.80	1.35 (0.50 to 2.20)	0.002	
	Class II	Study entry	19.95 ± 4.45	20.23 ± 4.44			
		Post-intervention	19.96 ± 3.66	22.70 ± 4.76	2.53 (1.71 to 3.34)	<0.001	
		Late pregnancy	20.31 ± 3.93	23.03 ± 4.43	2.08 (1.15 to 3.02)	<0.001	
	Class III	Study entry	20.02 ± 4.41	20.69 ± 4.73			
		Post-intervention	20.52 ± 4.30	22.85 ± 4.53	2.00 (0.74 to 3.25)	0.002	
		Late pregnancy	20.60 ± 4.65	23.65 ± 4.50	2.97 (1.50 to 4.44)	<0.001	
Total fat	Class I	Study entry	30.73 ± 5.57	30.98 ± 5.30			
(% energy)		Post-intervention	31.62 ± 4.87	30.44 ± 5.27	−1.10 (−1.94 to −0.26)	0.010	
		Late pregnancy	31.98 ± 5.02	30.84 ± 5.65	−0.96 (−1.98 to 0.05)	0.061	
	Class II	Study entry	31.34 ± 5.67	30.77 ± 5.05			
		Post-intervention	31.51 ± 5.32	30.14 ± 5.00	−1.05 (−2.12 to 0.02)	0.055	
		Late pregnancy	32.01 ± 5.07	30.47 ± 4.96	−1.36 (−2.48 to −0.24)	0.018	
	Class III	Study entry	31.16 ± 5.02	31.57 ± 5.84			
		Post-intervention	31.42 ± 5.42	31.07 ± 5.60	0.01 (−1.57 to 1.59)	0.992	
		Late pregnancy	32.09 ± 4.95	30.41 ± 5.94	−1.81 (−3.68 to 0.05)	0.057	
Saturated fat	Class I	Study entry	12.62 ± 3.13	12.49 ± 2.93			
(% energy)		Post-intervention	13.19 ± 2.85	12.12 ± 2.88	−0.87 (−1.32 to −0.42)	<0.001	
		Late pregnancy	13.43 ± 2.91	12.31 ± 3.01	−0.75 (−1.30 to −0.21)	0.007	
	Class II	Study entry	12.92 ± 2.97	12.31 ± 2.69			
		Post-intervention	13.19 ± 3.13	11.95 ± 2.76	−1.03 (−1.65 to −0.41)	0.001	
		Late pregnancy	13.64 ± 2.91	12.08 ± 2.51	−1.22 (−1.86 to −0.59)	<0.001	
	Class III	Study entry	12.60 ± 2.95	12.69 ± 2.95			
		Post-intervention	12.83 ± 2.97	12.13 ± 2.82	−0.53 (−1.36 to 0.31)	0.213	
		Late pregnancy	13.40 ± 2.76	12.36 ± 3.09	−1.12 (−2.08 to −0.16)	0.022	
Fibre	Class I	Study entry	13.78 ± 6.27	13.30 ± 5.16			
(g/day)		Post-intervention	12.64 ± 4.98	13.04 ± 4.88	0.63 (−0.20 to 1.47)	0.138	
		Late pregnancy	12.31 ± 5.61	13.19 ± 5.69	1.21 (0.13 to 2.30)	0.028	
	Class II	Study entry	13.80 ± 6.01	12.89 ± 5.50			
		Post-intervention	12.63 ± 5.96	13.25 ± 5.10	0.36 (−0.90 to 1.63)	0.570	
		Late pregnancy	12.15 ± 4.41	12.77 ± 5.45	0.70 (−0.34 to 1.74)	0.185	
	Class III	Study entry	12.81 ± 5.22	12.94 ± 5.35			
		Post-intervention	12.29 ± 4.53	14.81 ± 6.89	2.42 (0.69 to 4.16)	0.007	
		Late pregnancy	11.98 ± 6.34	13.43 ± 5.28	1.68 (−0.41 to 3.76)	0.115	
Physical Activity	Class I	Study entry	1404 (660–3252)	1386 (594–3492)			
MET		Post-intervention	1582.50 (693–4090)	1989.75 (924–5265)	0.27 (0.08 to 0.46)	0.006	
(min/week)		Late pregnancy	1386 (495–3413)	1485 (692.50–3702)	0.18 (−0.02 to 0.39)	0.084	
	Class II	Study entry	1506 (693–5163)	1386 (604.50–3478.50)			
		Post-intervention	1559 (685.50–3857.75)	1788.50 (693–4758.75)	0.08 (−0.14 to 0.30)	0.490	
		Late pregnancy	1432 (660–3375)	1539 (693–3150)	0.14 (−0.10 to 0.39)	0.250	
	Class III	Study entry	1233 (527.10–3865.50)	1386 (594–4455)			
		Post-intervention	1824 (495–5351)	2190 (986.50–5598)	0.25 (−0.80 to 0.58)	0.135	
		Late pregnancy	1485 (546–5119.50)	1846.50 (696.50–6021)	0.25 (−0.14 to 0.64)	0.209	
Walking	Class I	Study entry	280 (125–630)	280 (140–630)			
(min/week)		Post-intervention	290 (140–720)	420 (180–840)	0.32 (0.14 to 0.51)	0.001	
		Late pregnancy	300 (120–630)	315 (140–750)	0.22 (0.02 to 0.42)	0.031	
	Class II	Study entry	300 (150–840)	315 (140–630)			
		Post-intervention	360 (140–840)	332.50 (150–840)	−0.02 (−0.25 to 0.20)	0.843	
		Late pregnancy	300 (140–600)	360 (140–840)	0.15 (−0.10 to 0.40)	0.239	
	Class III	Study entry	240 (120–840)	300 (125–840)			
		Post-intervention	290 (100–1050)	420 (210–1260)	0.29 (−0.06 to 0.64)	0.102	
		Late pregnancy	280 (100–840)	420 (187.50–1050)	0.28 (−0.11 to 0.66)	0.160	

Data are mean ± SD for nutritional data and median (interquartile range) for physical activity data. ^a^ Study entry data collected at 15^+0^–18^+6^ weeks’ gestation, data post-intervention collected at 27^+0^–28^+6^ weeks, data at late gestation taken at 34^+0^–36^+0^ weeks. ^b^ *p* value to assess difference in nutrition and physical activity post-intervention and in late pregnancy, between equivalent obesity classes, randomised to standard care and intervention, calculated using ANCOVA, adjusting for values at study entry. *p* < 0.05 taken as significant ^c^ *p* value to assess for an interaction of obesity class and intervention group on nutritional and physical activity outcomes. For nutritional outcomes, the following are the BMI = 30.0–34.9 kg/m^2^ control group observations: *n* = 269, *n* = 243, *n* = 193 at baseline, post-intervention and in late pregnancy, respectively. BMI = 35.0–39.9 kg/m^2^ control group observations: *n* = 200, *n* = 181, *n* = 149. BMI = +40.0 kg/m^2^ control group observations: *n* = 101, *n* = 90, *n* = 75. BMI = 30.0–34.9 kg/m^2^ intervention group observations: *n* = 295, *n* = 235, *n* = 180. BMI = 35.0–39.9 kg/m^2^ intervention group observations: *n* = 180, *n* = 132, *n* = 109. BMI = +40.0 kg/m^2^ intervention group observations: *n* = 99, *n* = 68, *n* = 58. For physical activity outcomes, the following are the BMI = 30.0–34.9 kg/m^2^ control group observations: *n* = 321, *n* = 278, *n* = 245. BMI = 35.0–39.9 kg/m^2^ control group observations: *n* = 241, *n* = 204, *n*= 175. BMI = +40.0 kg/m^2^ control group observations: *n* = 116, *n* = 106, *n* = 89. BMI = 30.0–34.9 kg/m^2^ intervention group observations: *n* = 346, *n* = 290, *n* = 235. BMI = 35.0–39.9 kg/m^2^ intervention group observations: *n* = 212, *n* = 168, *n* = 139. BMI = +40.0 kg/m^2^ intervention group observations: *n* = 125, *n* = 101, *n* = 84.

**Table 3:** Maternal and neonatal outcomes by intervention group and obesity class.

	Obesity Class	Standard Care	Intervention	Effect of Intervention		*p* ^d^	*p* ^e^
				Odds Ratio (95% CI)	Mean Difference (95% CI)		
Fasting blood glucose	Class I	4.64 ± 0.52 *n* = 320	4.59 ± 0.51 *n* = 330		−0.05 (−0.13 to 0.03)	0.260	
(mmol/L)	Class II	4.71 ± 0.55 *n* = 224	4.75 ± 0.68 *n* = 194		0.02 (−0.10 to 0.14)	0.715	
	Class III	4.87 ± 0.69 *n* = 121	4.84 ± 0.63 *n* = 116		−0.03 (−0.20 to 0.15)	0.760	
1 h blood glucose	Class I	7.84 ± 1.94 *n* = 301	7.69 ± 2.09 *n* = 306		−0.14 (−0.45 to 0.18)	0.391	
(mmol/L)	Class II	8.12 ± 2.16 *n* = 201	8.23 ± 2.11 *n* = 183		0.09 (−0.34 to 0.52)	0.680	
	Class III	8.37 ± 2.31 *n* = 108	7.93 ± 1.95 *n* = 104		−0.44 (−1.03 to 0.15)	0.145	
2 h blood glucose	Class I	5.83 ± 1.38 *n* = 320	5.86 ± 1.51 *n* = 330		0.06 (−0.16 to 0.28)	0.590	
(mmol/L)	Class II	6.00 ± 1.49 *n* = 223	6.17 ± 1.62 *n* = 194		0.16 (−0.15 to 0.46)	0.315	
	Class III	6.15 ± 1.69 *n* = 121	5.92 ± 1.35 *n* = 115		−0.20 (−0.59 to 0.20)	0.332	
Gestational weight gain	Class I	5.55 ± 2.58 *n* = 318	5.47 ± 2.94 *n* = 328		−0.07 (−0.49 to 0.36)	0.757	
to 27^+0^–28^+6^ weeks days	Class II	5.36 ± 2.93 *n* = 223	4.81 ± 2.80 *n* = 193		−0.52 (−1.08 to 0.04)	0.071	
(kg)	Class III	4.94 ± 2.95 *n* = 123	3.83 ± 2.81 *n* = 116		−1.12 (−1.88 to −0.37)	0.004	
Interaction							0.033
Gestational weight gain total ^a^ (kg)	Class I	8.04 ± 4.33 *n* = 277	8.03 ± 4.50 *n* = 265		−0.11 (−0.84 to 0.61)	0.762	
	Class II	7.63 ± 4.44 *n* = 190	6.86 ± 4.24 *n* = 164		−0.75 (−1.67 to 0.17)	0.111	
	Class III	7.43 ± 4.98 *n* = 100	5.44 ± 4.87 *n* = 97		−1.87 (−3.29 to −0.47)	0.009	
Interaction							0.023
Maternal sum of skinfold thicknesses	Class I	115.22 ± 21.77 *n* = 316	110.98 ± 20.89 *n* = 328		−3.90 (−7.19 to−0.60)	0.021	
at 27–28 weeks + 6 days ^c^	Class II	129.26 ± 21.65 *n* = 223	130.96 ± 23.98 *n* = 191		2.10 (−2.35 to 6.56)	0.354	
(mm)	Class III	152.19 ± 25.92 *n* = 122	148.79 ± 27.75 *n* = 113		−3.25 (−10.28 to 3.77)	0.363	
Maternal sum of skinfold thicknesses	Class I	113.85 ± 22.64 *n* = 274	110.04 ± 20.55 *n* = 263		−3.10 (−6.77 to 0.57)	0.098	
at 34–36 weeks + 0 days ^c^	Class II	128.05 ± 24.03 *n* = 188	127.57 ± 22.67 *n* = 162		−0.52 (−5.45 to 4.42)	0.837	
(mm)	Class III	149.52 ± 25.63 *n* = 99	144.57 ± 25.42 *n* = 95		−4.54 (−12.03 to 2.96)	0.234	
Gestational diabetes ^b^	Class I	67/320 (21%)	63/330 (19%)	0.92 (0.62 to 1.38)		0.699	
	Class II	60/224 (27%)	65/194 (34%)	1.32 (0.85 to 2.04)		0.216	
	Class III	48/121 (40%)	35/116 (30%)	0.65 (0.37 to 1.15)		0.141	
Pre-eclampsia	Class I	10/362 (3%)	14/375 (4%)	1.46 (0.63 to 3.36)		0.379	
	Class II	7/256 (3%)	8/239 (3%)	1.34 (0.46 to 3.92)		0.592	
	Class III	10/134 (7%)	5/141 (4%)	0.39 (0.12 to 1.31)		0.128	
Caesarean section	Class I	123/370 (33%)	134/380 (35%)	1.07 (0.78 to 1.46)		0.690	
	Class II	80/253 (32%)	72/240 (30%)	0.91 (0.61 to 1.35)		0.623	
	Class III	71/134 (53%)	64/143 (45%)	0.70 (0.43 to 1.15)		0.160	
Large for gestational age ≥ 90th	Class I	25/370 (7%)	38/380 (10%)	1.50 (0.88 to 2.56)		0.140	
(customised birthweight centiles)	Class II	17/253 (7%)	18/240 (8%)	1.12 (0.55 to 2.28)		0.746	
	Class III	20/134 (15%)	15/143 (10%)	0.69 (0.33 to 1.44)		0.324	
Small for gestational age ≤ 10th	Class I	33/370 (9%)	53/380 (14%)	1.70 (1.06 to 2.71)		0.026	
(customised birthweight centiles)	Class II	28/253 (11%)	29/240 (12%)	1.15 (0.65 to 2.03)		0.627	
	Class III	18/134 (13%)	15/143 (10%)	0.79 (0.37 to 1.67)		0.529	
Birthweight ≥ 4.0(kg)	Class I	50/370 (14%)	59/380 (16%)	1.20 (0.79 to 1.82)		0.396	
	Class II	32/253 (13%)	27/240 (11%)	0.88 (0.50 to 1.54)		0.656	
	Class III	23/134 (17%)	19/143 (13%)	0.68 (0.34 to 1.36)		0.282	

Data are mean ± SD, or number of women, or neonates/total (%) for maternal and neonatal outcomes, respectively. **^a^** Gestational weight gain was calculated using estimated weight pre-pregnancy weight. **^b^** Gestational diabetes was diagnosed using IADPSG criteria. **^c^** Calculated by addition of biceps, triceps, suprailiac and subscapular skinfold thicknesses. **^d^** *p* value to assess for difference in maternal/neonatal outcome between obesity class randomised to intervention and standard care, assessed using linear and logistic regression, adjusting for potential cofounders: index of multiple deprivation, parity, age, ethnicity and years in full-time education. *p* < 0.05 taken as significant. **^e^** *p* value to assess for the effect of an interaction of obesity class and intervention group on maternal and neonatal outcomes; only significant results are tabulated.

## Supplementary Materials

The following are available online at https://www.mdpi.com/1660-4601/17/13/4712/s1, Table S1: Health behaviours of pregnant women with obesity by obesity class at study entry.
