# Supplementary material for: The UK Pregnancies Better Eating and Activity Trial (UPBEAT); Pregnancy Outcomes and Health Behaviours by Obesity Class
Source: Int J Environ Res Public Health. 2020 Jun 30;17(13):4712. doi: 10.3390/ijerph17134712 (PMC7370059; doi:10.3390/ijerph17134712)
Supplement: Supplementary file 1 [file ijerph-17-04712-s001.pdf]

Table S1. Health behaviours of pregnant women with obesity by obesity class at study entry.

	Obesity Class			
**Dietary intake at study entry** [a]	**Class I** (***n*** **= 564)**	**Class II** (***n*** **=380)**	**Class III** (***n*** **= 200)**	***p*** [b]
Total energy (Kcal)	1852.31 ± 603.32	1824.73 ± 592.40	1831.17 ± 631.97	0.772
Glycaemic index (0-100)	56.83 ± 4.15	56.70 ± 4.07	57.03 ± 3.48	0.647
Glycaemic load (per day)	140.31 ± 55.35	135.37 ± 50.93	134.96 ± 53.27	0.276
Carbohydrate (% energy)	49.66 ± 7.53	49.00 ± 7.29	48.48 ±7.21	0.117
Protein (% energy)	19.63 ± 4.36	20.08 ± 4.45	20.36 ± 4.57	0.091
Total fat (% energy)	30.86 ± 5.43	31.07 ± 5.38	31.36 ± 5.43	0.517
Saturated fat (% energy)	12.55 ± 3.03	12.63 ± 2.85	12.65 ± 2.94	0.880
Fibre (g/day)	13.53 ± 5.72	13.37 ± 5.78	12.87 ± 5.27	0.371
Physical activity at baseline	30–34.9 (*n* = 667)	35.0–39.9 (*n* = 435)	≥40.0 (*n* = 241)	
Walking (minutes/week)	280 ± 490	315 ± 480	280 ± 720	0.349
MET [c] (minutes/week)	1386 ± 2715	1485 ± 3261	1320 ± 3655.5	0.405

Nutritional data are mean ± SD, physical activity data are median ± IQR [a] Study entry data collected at 15^{+0}–18^{+6} weeks' gestation, [b] *p*: differences between nutritional variables and the log transformation of physical activity measures by obesity class, assessed using ANOVA. $p < 0.05$ taken as significant [c] MET = metabolic equivalent of task, is defined as the energy expenditure ratio of activity to rest
